# Study to Assess the Clinical and Sociodemographic Profile of Measles in Children Admitted to a Tertiary Care Center: A Retrospective Study

**DOI:** 10.7759/cureus.65843

**Published:** 2024-07-31

**Authors:** Sagar V, Shivaprakash C Sosale, Sahana Devadas, Sujatha P, Gayathri Devi Chinnappa

**Affiliations:** 1 Pediatrics, Bangalore Medical College and Research Institute, Bangalore, IND

**Keywords:** infectious disease, immunity, prevention, immunization, measles

## Abstract

Background

Measles remains a significant contributor to illness and death among young children, despite the presence of a safe and efficacious vaccine. One of the most highly transmissible viruses affecting humans is measles, which can be almost entirely prevented through vaccination. The recent surge in measles cases worldwide has been particularly notable in India. A total of 172 confirmed outbreaks of measles were recorded between October 2021 and September 2022, resulting in 12,589 cases. Measles, being highly contagious, is transmitted through the respiratory route. The disease affects 75 to 90% of susceptible household contacts, reaching peak infectivity three days before the rash emerges. The recent measles outbreak observed since January 2023 holds significance, as Bangalore district alone has reported around 500 cases. Therefore, the current study aims to evaluate the clinical and sociodemographic characteristics of measles in pediatric patients.

Methods

This was a retrospective study conducted over seven months. Data pertaining to demographics such as age, gender, parental occupation, parental educational background, and housing situation were gathered. Details regarding the child's immunization status, the onset of fever, and the onset of rash were also documented. Descriptive data was represented using mean, standard deviation, and percentage or proportion.

Results

Over the study period of seven months, 53 children were admitted due to measles. The majority of the individuals were female, accounting for 28 cases (52.8%). The typical clinical manifestations of measles such as cough, coryza, and conjunctivitis were documented in 45 (84.90%), 43 (81.13%), and 32 (60.37%) children, respectively. Specifically, two (3.77%), 14 (26.41%), 20 (37.73%), and 17 (32.07%) individuals were categorized as belonging to the upper middle, lower middle, upper lower, and lower socioeconomic strata. Six children in the nine- to 12-month age range had not received the measles-rubella (MR) vaccination. Thirteen (54.16%) out of 24 children aged one to five years had not received a single dose of the vaccine, while the children who had received at least one dose did not experience any complications. Among children aged less than nine months, nine to 12 months, and more than 12 months, three (100%), two (66.6%), and three (75%) respectively, experienced complications. All the patients recovered completely and were discharged from the hospital.

Conclusion

Measles is re-emerging as an infectious disease among children, as evidenced by the current outbreak. Study indicates that measles tends to impact infants and under five children more frequently. Through prompt and suitable medical intervention, all affected children experienced full recovery. It was observed that immunized children had fewer complications in comparison to their non-immunized counterparts. Acquiring a thorough understanding of the clinical profile of the illness is crucial for policymakers in developing essential guidelines for measles immunization coverage.

## Introduction

Measles remains a significant contributor to illness and death among young children, despite the presence of a safe and efficacious vaccine [1]. One of the most highly transmissible viruses affecting humans is measles, which can be almost entirely prevented through vaccination [2].

As indicated in a 2021 report by the World Health Organization, the global count of measles-related deaths stood at 128,000, with an approximate nine million cases reported. The recent surge in measles cases worldwide has been particularly notable in India. A total of 172 confirmed outbreaks of measles were recorded between October 2021 and September 2022, resulting in 12,589 cases.

Measles commences with an escalating body temperature (reaching 39-40.5 °C), accompanied by cough, coryza, and conjunctivitis following an incubation period of eight to 12 days [3,4]. The appearance of the rash typically initiates on the facial and neck regions, manifesting as distinct, erythematous patches varying in diameter from 3 to 8 mm. This rash endures for a duration of three to seven days and subsequently vanishes in a manner similar to its onset. Children suffering from malnutrition often exhibit heightened desquamation [5,6]. Koplik's spots usually emerge a day prior to the onset of the rash and persist for a period of two to three days [7]. The presence of Koplik's spots has been documented in 60% to 70% of individuals diagnosed with measles [8].

Measles, being highly contagious, is transmitted through the respiratory route. The disease affects 75 to 90% of susceptible household contacts, reaching peak infectivity three days before the rash emerges [9,10]. Complications such as pneumonia, croup, and encephalitis often lead to fatalities, with encephalitis representing the most severe long-term outcome.

The estimated coverage for the initial dose of a measles- and rubella-containing vaccine rose from 68% to 89% in 2021. Similarly, there was a notable increase in the estimated coverage for the second dose of a measles-containing vaccine, climbing from 27% to 82% during the same year [11]. Over the period of 2017-2021, there was a 62% decrease in measles incidence and a 48% decrease in rubella incidence.

The recent measles outbreak observed since January 2023 holds significance, as Bangalore district alone has reported around 500 cases. Therefore, the current study aims to evaluate the clinical and sociodemographic characteristics of measles in pediatric patients.

## Materials and methods

A retrospective study was carried out from January 2023 to July 2023 at the pediatrics department of Vani Vilas Hospital. All pediatric patients admitted to Vani Vilas Hospital with clinically consistent measles during study period were considered for inclusion in the research. Patients with symptoms such as fever and rash that did not align with the clinical presentation of measles, or those with non-maculopapular rashes, were deemed ineligible for participation in the study.

All patients who were admitted to the Department of Pediatrics at Bangalore Medical College and Research Institute (BMCRI) and met the specified inclusion criteria were enrolled in the research. Data pertaining to demographics such as age, gender, parental occupation, parental educational background, and housing situation were gathered. Details regarding the child's immunization status, the onset of fever, and the onset of rash were also documented. Throat swabs and serum samples were obtained from all patients exhibiting clinically compatible symptoms. The evaluation methods employed included reverse transcription polymerase chain reaction (RT-PCR) analysis of throat swab samples and estimation of serum IgM measles antibodies. The outcomes were assessed based on the length of hospitalization, necessity for intensive care unit (ICU) intervention, occurrence of complications, and patients' immunization statuses. Anemia status of children was assessed based on WHO cut-off for anemia based on age and sex group (Table 1) [12].

**Table 1:** WHO cut off of anemia based on age and sex group Hemoglobin is measured in g/dL

Age/ Sex group	No anemia (g/dL)	Mild (g/dL)	Moderate (g/dL)	Severe (g/dL)
Children 6-59 months	>11	10-10.9	7-9.9	<7
Children 5-11 years	>11.5	11-11.4	8-10.9	<8
Children 12-14 years	>12	11-11.9	8-10.9	<8
Non pregnant women (15 years and above)	>12	11-11.9	8-10.9	<8
Men (15 years and more)	>13	11-12.9	8-10.9	<8

Complications may encompass conditions such as blindness, encephalitis (a viral-induced inflammation leading to cerebral edema and possible neurological impairment), acute gastroenteritis and consequent fluid imbalance, otitis media, and acute respiratory distress syndrome including pneumonia.

SPSS version 21 (IBM Corp., Armonk, NY, USA) was used to perform the statistical analysis. Data was entered in an Excel spreadsheet (Microsoft, Redmond, WA, USA). Descriptive statistics of the explanatory and outcome variables were calculated by mean, standard deviation for quantitative variables, frequency and proportions for qualitative variables.

## Results

During the study period, a total of 53 children were admitted with measles. The presence of measles was confirmed in two (3.7%) children through positive RT-PCR on a throat swab, in 27 (50.9%) cases through positive IgM antibody, and in two (3.7%) cases through positivity for both throat swab and serum IgM antibodies. Among all cases, 24 (45.3%) were clinically compatible with measles. Three cases declined sample collection but exhibited clinical compatibility.

The sociodemographic profile and clinical characteristics of the cases are detailed in Table 2. Examination of Table 2 reveals that infants comprised the majority of cases (N=18, 33.9%) in the study, with the youngest infant being merely two months old.

**Table 2:** Sociodemographic profile and clinical features N=53 Socioeconomic status assessed by Modified Kuppuswamy Classification

Age	N	%
<9 months	9	16.9
9-12 months	9	16.9
1-5yrs	24	45.2
>5yrs	11	20.7
Gender		
Males	25	47.1
Females	28	52.8
Classical symptoms		
Cough	45	84.9
Coryza	43	81.1
Conjunctivitis	32	60.3
Nutritional status		
Severe Malnutrition	4	7.5
Moderate Acute Malnutrition	4	7.5
Anemia	18	33.9
Socioeconomic status		
Upper Middle	2	3.7
Lower Middle	14	26.4
Upper Lower	20	37.7
Lower	17	32.0

A total of nine cases (16.9%) were recorded in individuals under nine months of age, while another nine cases (16.9%) were reported in children aged nine to 12 months. The largest proportion of cases, constituting 24 children (45.2%), fell within the one to five years age group. Additionally, 11 children (20.7%) were identified as being over five years old. The majority of the individuals were female, accounting for 28 cases (52.8%).

The typical clinical manifestations of measles such as cough, coryza, and conjunctivitis were documented in 45 (84.90%), 43 (81.13%), and 32 (60.37%) children, respectively. A rash was observed by the fourth day following the onset of fever in 33 (62.2%) children, with five children (9.4%) displaying a rash on the initial day of fever. Among the cases, 22 (41.5%) had a history of contact, with 14 children developing rashes within a week of exposure. Malnutrition was present in eight (15.0%) cases, while 18 (33.9%) cases had anemia.

Specifically, two (3.77%), 14 (26.41%), 20 (37.73%), and 17 (32.07%) individuals were categorized as belonging to the upper middle, lower middle, upper lower, and lower socioeconomic strata according to the Modified Kuppuswamy Classification. It is of significance to highlight that more cases were observed among individuals in the upper lower and lower socioeconomic status.

**Figure 1:**
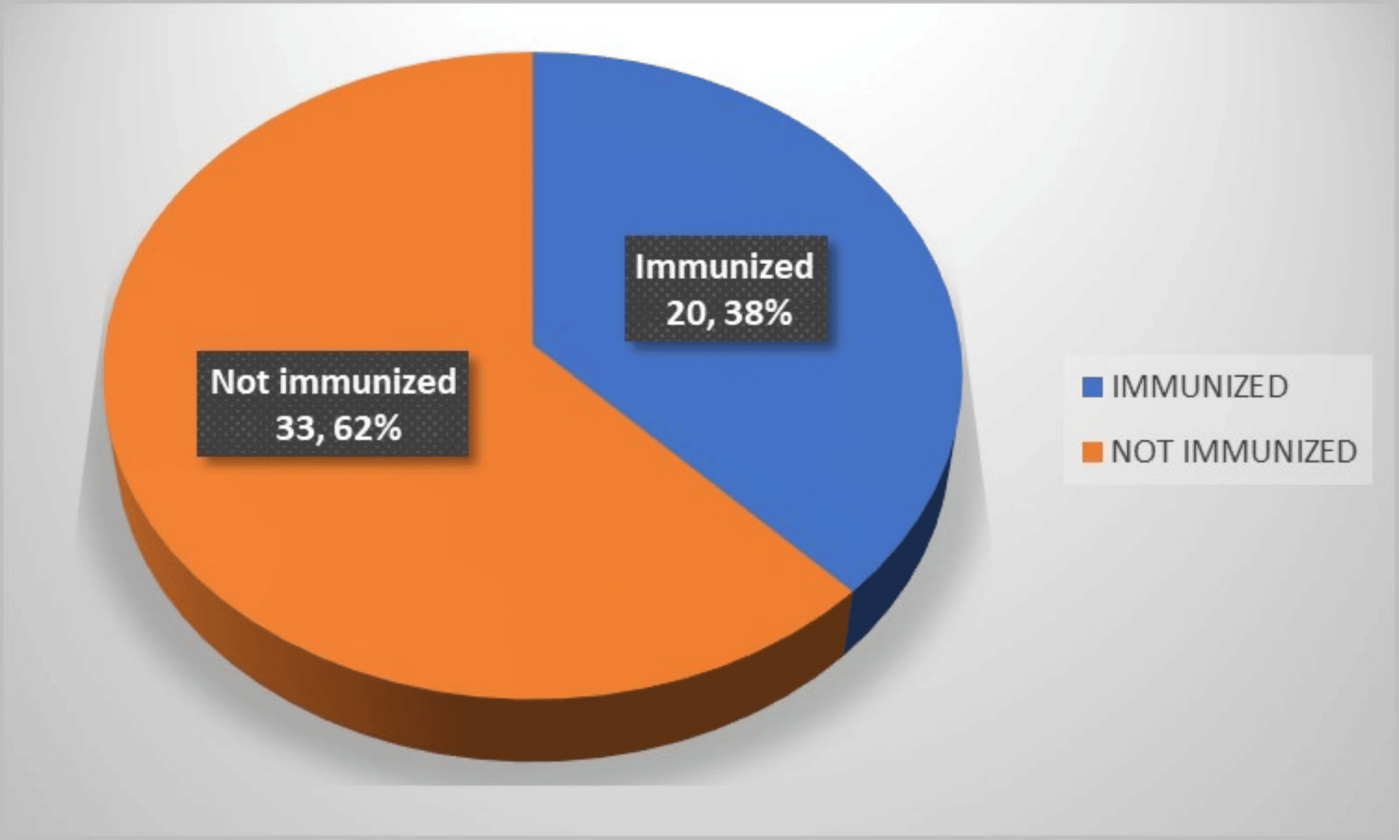
Overall immunization status N=53

Out of the total number of cases, 20 cases, equivalent to 37.7%, had been administered with a minimum of one dose of the measles-rubella (MR) vaccine, while the remaining 33 cases, representing 62.2% of the total, had not received immunization against measles (Figure 1).

A total of 33 (62.2%) children who did not receive immunization against measles were reported to have acquired the infection, as opposed to 14 (26.4%) children who had received a single dose and six (11.3%) children who had completed both doses of the measles-containing vaccine.

In the nine to 12 months age bracket, there were six children who had not been administered the MR vaccine, with five instances (83.3%) being confirmed as measles through laboratory testing. Among children aged between one and five years, 13 out of 24 children (54.1%) had not received any dosage of the measles-containing vaccine. Furthermore, three out of 24 children (12.5%) within the one to five years age group developed measles despite being given two doses of the vaccine. Moving on to children aged five years and above, five out of 11 cases (45.4%) had not been vaccinated against measles. Within this group, three out of 11 children (27.3%) had received one vaccine dose, while an equal number of children, three out of 11 (27.3%), who had completed both doses, still contracted measles (Table 3).

**Table 3:** Immunization status of all cases N=53

	<9 months(N)	%	9-12 months(N)	%	1-5years(N)	%	>5years(N)	%	Total	%
1 Dose	Not applicable	0	3	33.3	8	33.3	3	27.2	14	26.4
2 Doses	Not applicable	0	Not applicable	0	3	12.5	3	27.2	6	11.3
Not immunized	9 (100%)	100	6	66.6	13	54.1	5	45.4	33	62.2
TOTAL	9	100	9	100	24	100	11	100	53	100

Among children younger than nine months, all individuals were found to be without immunization, as the primary dose is typically administered at nine months of age. Among the lab confirmed measles in the age group of nine to 12 months, five children (62.5%) were identified as not having received immunization, while among children aged one to five years, seven children (77.7%) were lacking immunization against measles. In total, 20 children (68.9%) diagnosed with lab-confirmed measles had not been administered even a single dose of the measles-containing vaccine (Table 4).

**Table 4:** Immunization status of lab-confirmed cases N=29

	<9 Months(N)	%	9-12 Months(N)	%	1-5years(N)	%	>5years(N)	%	Total	%
1 Dose	Not applicable	0	3	37.5	1	11.1	2	50	6	20.6
2 Doses	Not applicable	0	Not applicable	0	1	11.1	2	50	3	10.3
Not immunized	8	100	5	62.5	7	77.7	0	0	20	68.9
Total	8	100	8	100	9	100	4	100	29	100

Table 5 illustrates that three (30%) children experienced pneumonia, four (40%) cases manifested acute diarrheal disease, two (20%) cases exhibited febrile seizures, and one (10%) case presented with ASOM as complications. Within the group of three children who developed pneumonia, two were infants under nine months of age, both of whom tested positive for measles. It is noteworthy to highlight that out of all 14 children who had received at least a minimum of one vaccine dose, no adverse effects were observed, and the illnesses were characterized by mild severity and brief duration.

**Table 5:** Complications N=10 ASOM - Acute Suppurative Otitis Media

	<9 Months(N)	%	9-12 Months(N)	%	>12 Months(N)	%	Total	%
Pneumonia	2	66.6	0	0	1	25	3	30
Acute gastroenteritis	1	33.3	2	66.6	1	25	4	40
ASOM	0	0	0	0	1	25	1	10
Febrile seizures	0	0	1	33.3	1	5	2	20
Total	3	100	3	100	4	100	10	100

Table 6 illustrates the correlation between the occurrence of complications and the immunization status. Among children aged less than nine months, nine to 12 months, and more than 12 months, three (100%), two (66.6%), and three (75%) respectively, who were unimmunized, experienced complications. Complications were noted commonly in infants and unimmunized population.

**Table 6:** Immunization status in children with complications N=10

	<9 Months(N)	%	9-12 Months(N)	%	>12 Months(N)	%	Total	%
Immunized	0	0	1	33.3	1	25	2	20
Not immunized	3	100	2	66.6	3	75	8	80
Total	3	100	3	100	4	100	10	100

However, it is worth mentioning that in our investigation, it was observed that a total of 53 children were discharged successfully without any fatalities. It is noteworthy to highlight that nine children (16.9%) necessitated stays in the ICU, with an average length of five days. The remaining 44 children (83%) received symptomatic management while hospitalized.

## Discussion

History of incomplete immunization, low socioeconomic status, overcrowding and contact history, have been observed as risk factors for measles. Furthermore, factors like young age and malnutrition have been identified as independent contributors to the risk of contracting measles. The research conducted revealed that the average age of the children involved was approximately 2.5 years old, with over a third having been in contact with measles, and about two-thirds having incomplete or no immunisation. A significant portion, around 70%, of the cases belonged to socio-economic classes 4 and 5, while 15% were found to be suffering from malnutrition. It was noted that young age, malnutrition, and lack of proper immunization were independent risk factors associated with the need for ICU admission and the development of complications.

In 2018, a retrospective study was carried out by Mehta and colleagues at a tertiary care center in Gujarat to examine the clinical characteristics, complications, and outcomes of measles in children. Throughout the study duration, 42 children with fever and rash were confirmed to have measles based on IgM-positive antibody titres. Half of the cases did not receive any vaccinations, with a higher prevalence among males than females. The majority of patients (78.57%) resided in urban slum areas, and most cases (81%) occurred in late winter and early spring. Pneumonia emerged as the most frequent complication of measles, affecting 31 out of the 42 patients. Fortunately, all patients made a full recovery and were discharged from the hospital [13]. In our study females were affected more frequently compared to males and 54.7% were lab-confirmed measles. Among the complications, acute diarrheal disease was more common than pneumonia. 69.8% of children belonged to lower socioeconomic status and all children were discharged from the hospital with full recovery.

Monfort et al. conducted a study on a measles outbreak in Barcelona, focusing on the clinical and epidemiological aspects. The research indicated that 23.6% of patients had been vaccinated against measles, and 55.3% had previous exposure to confirmed cases. The most commonly reported complications were pneumonia (15.3%) and acute otitis media (20.4%). Approximately 23.5% of patients required hospitalization. Their study highlighted that a considerable proportion of infants, specifically 33.96% of cases, were affected. In children, atypical manifestations of measles are prevalent, and the presence of maternal antibodies can influence the clinical features. Notably, nine out of 18 infants developed measles before the recommended age of nine months for the initial measles-containing vaccine dose in India. Unlike immunity from natural infection, early decline in maternal antibodies post-vaccination can trigger immunity [14-16]. Similar observations were noted in our study where infants constituted 33.9% of total cases with significant number of infants below nine months of age being affected. Despite the relatively low incidence of measles complications in our study, infants face a higher risk of severe outcomes such as bronchopneumonia and diarrhea.

Prospective observational research conducted by Raote et al. examined the clinical characteristics of 150 hospitalized children with measles. Their findings revealed that early PEM grades were linked to modest challenges, while the well-nourished cohort did not encounter any issues. Conversely, children suffering from severe malnutrition faced more frequent and severe complications [17]. It is noteworthy that measles significantly contributes to the onset of malnutrition, and reciprocally, it can lead to blindness in preschoolers due to vitamin A deficiency [18]. The nutritional status of individuals also significantly impacts measles susceptibility. Our study revealed that 18 out of 53 cases suffered from anemia, while eight children were experiencing malnutrition. Additionally, measles contributes significantly to the onset of blindness in preschool children due to vitamin A deficiency [18].

In 2006, a cross-sectional study conducted at New York University by Krugman et al. unveiled that detectable antibodies against the measles virus emerged by the 12th day following the initial infection, with peak antibody titers occurring between the 21st and 28th days. The antibody levels remained adequate for a minimum of four years, effectively preventing the measles infection. Exposure to the measles virus frequently resulted in an asymptomatic infection and a secondary response to boost antibody levels when they declined to minimal or undetectable levels; during such instances, antibodies became detectable by the seventh day and reached peak levels by the 12th day. Consequently, it can be inferred that enduring immunity is provided by a single encounter with measles and a solitary administration of live measles virus vaccination [19].

Atypical manifestations were frequently observed in infant measles cases, and the clinical presentation might have been influenced by the presence of maternal antibodies, as indicated in a retrospective study conducted by Sindhu and colleagues at a tertiary care center in Kerala [14]. This could potentially be attributed to the rapid decline of maternal antibodies post-vaccination compared to immunity acquired from natural infection [15,16]. The documentation of immunization records holds significant importance in the process of medical history acquisition. Diligent surveillance is deemed necessary for populations at high risk such as unvaccinated children and infants. Findings from our research indicated that a considerable proportion (33%) of children had not been administered with any dose of measles-containing vaccine, leading to complications observed in 10 unvaccinated children (18.8%).

Timely recognition of the typical manifestations of measles, expeditious intervention, and appropriate isolation of individuals contribute significantly to interrupting the spread of the disease. This goal is facilitated through a comprehensive understanding of the characteristic clinical profile associated with measles. Vigilance for potential complications in non-immunized pediatric patients, coupled with effective intensive care and vigilant surveillance, plays a crucial role in diminishing mortality rates attributable to measles.

Limitations of the study include its confined timeframe and single-center study. A considerable number of cases were documented solely in the Bangalore district. A comprehensive study is imperative to evaluate the efficacy of measles immunization and to promote awareness aimed at enhancing vaccine coverage.

## Conclusions

Measles is re-emerging as an infectious disease among children, as evidenced by the current outbreak. Study indicates that measles tends to impact infants and under five children more frequently. Through prompt and suitable medical intervention, all affected children experienced full recovery without any long-term consequences. It was observed that immunized children had fewer complications in comparison to their non-immunized counterparts. Acquiring a thorough understanding of the clinical profile of the illness is crucial for policymakers in developing essential guidelines for measles immunization coverage. Subsequent research endeavors focusing on the efficacy of interventions and preventive strategies against measles are warranted to prevent future outbreaks.

## Appendices

Annexure 1

**Table 7:** Case Record Form

Sl Number	Components
1	Name
2	Age
3	Sex
4	Address
5	Chief Complaints
6	Past History
7	Family History
8	Birth History
9	Nutritional History
10	Immunization History
11	Developmental History
12	Socioeconomic History
13	Vitals
14	Anthropometry
15	Head To Toe Examination
16	Systemic Examination
17	Diagnosis
18	Investigations
19	Complications

Annexure 2

Case Definition of Measles

Centers for Disease Control and Prevention (CDC) definition of clinically compatible measles:

A clinical case of measles is

1) A generalized maculopapular rash lasting 3 or more days

2) Temperature of 38.3 C (101 F) or greater

3) One of the following: cough, coryza, conjunctivitis.
